# Risk Factors and Timing of Additional Surgery after Noncurative ESD for Early Gastric Cancer

**DOI:** 10.1155/2022/3421078

**Published:** 2022-06-21

**Authors:** Kaipeng Duan, Dongbao Li, Dongtao Shi, Jie Pei, Jiayu Ren, Weikang Li, Anqi Dong, Tao Chen, Jin Zhou

**Affiliations:** ^1^Department of General Surgery, The First Affiliated Hospital of Soochow University, Suzhou, Jiangsu 215006, China; ^2^Department of Gastroenterology, The First Affiliated Hospital of Soochow University, Suzhou, Jiangsu 215006, China

## Abstract

**Background:**

Patients with early gastric cancer undergoing noncurative endoscopic submucosal dissection (ESD) have a risk of tumor recurrence and metastasis, and some patients need additional surgery. The purpose of this study was to explore the risk factors of cancer residue and lymph node (LN) metastasis after noncurative ESD for early gastric cancer and to compare the short outcome of early and delayed additional surgery.

**Methods:**

The clinicopathological characteristics of 30 early gastric cancer patients who received noncurative ESD and additional surgery were studied retrospectively. Multivariable regression was utilized to examine the independent risk factors for residual cancer and LN metastasis. Receiver operating characteristic curve was used to analyze the multivariable model's predictive performance. Furthermore, the perioperative safety and radical tumor performance of early surgery (≤30 days, *n* = 11), delayed surgery (>30 days, *n* = 11) after ESD, and upfront surgery (*n* = 59) were compared.

**Results:**

Multivariable regression showed that diffuse type of Lauren classification, submucosal invasion, and positive human epidermal growth factor receptor-2 (HER-2) were risk factors for residual cancer. Undifferentiated carcinoma, vascular invasion, and positive vertical margin were risk factors for LN metastasis. The area under the curve (AUC) of the multifactor model predicting cancer residue and LN metastasis was 0.761 and 0.792, respectively. The early surgery group experienced higher intraoperative blood loss and a longer operation time than the delayed surgery and upfront surgery groups. There was no significant difference in the number of LN dissections, LN metastasis rate, and postoperative complications among the three groups.

**Conclusion:**

Diffuse type of Lauren classification, submucosal invasion, and positive HER-2 are risk factors for residual cancer, while undifferentiated carcinoma, vascular invasion, and positive vertical margin are risk factors for LN metastasis. Delayed additional surgery after ESD (>30 days) has higher intraoperative safety, without affecting the radical resection in early gastric cancer patients.

## 1. Introduction

At present, gastric cancer is still one of the diseases seriously endangering human health. The treatment effect of advanced gastric cancer and early gastric cancer differs greatly, with the 5-year survival rate of the former being less than 30% while the latter exceeding 90% [1, 2]. Therefore, early detection and standardized treatment are the keys to improving the long-term outcome of gastric cancer patients.

Endoscopic submucosal dissection (ESD) is an important treatment for early gastric cancer. Japanese guidelines for the treatment of gastric cancer recommend ESD as the preferred treatment for early gastric cancer with a low risk of lymph node (LN) metastasis, and the eCura evaluation system is used to judge its radical performance [3]. The noncurative ESD mainly includes two types: one is nonwhole resection or positive horizontal resection margin (eCura-1) and the other is associated with high-risk factors for LN metastasis (eCura-2) [3–5]. For the former, individualized treatment can be adapted according to the specific situation, including re-ESD treatment [6], additional surgery [7], or close follow-up [8]. For the latter, additional surgery is recommended [9]. However, in practice, it was common that neither cancer residue nor LN metastasis was found in cases receiving additional surgery after noncurative ESD.

There is still some controversy on how to choose remedial measures for cases with noncurative ESD. Accurate evaluation of residual cancer and LN metastasis after ESD is the main basis for determining remedial measures. If corrective surgery is chosen, there is no agreement on the ideal time to perform it. Therefore, in the present study, we hypothesized that the clinicopathological features of the ESD tissue can be used to predict cancer residual and LN metastasis in patients with early gastric cancer. Meanwhile, the timing of additional surgery may affect the perioperative outcome.

## 2. Materials and Methods

The clinical and pathological data of 89 patients with early gastric cancer who received surgical treatment in the First Affiliated Hospital of Soochow University from July 2016 to June 2019 were collected.

Patients who have confirmed early gastric cancer by postoperative pathology, namely, the cancer invasion was limited to the mucosa and submucosa, and preoperative CT, MRI, and other examinations without signs of distant metastasis were included in the study. Patients who underwent emergency surgery due to bleeding or perforation caused by ESD treatment, or with a history of endoscopic gastric surgery or upper abdominal surgery, or with heart, lung, liver, kidney, and other organ dysfunction and abnormal coagulation function before surgery, or with other types of malignant tumors, or with incomplete clinical and pathological data were excluded from the study. This study was approved by the hospital ethical committee (20190511003).

Among them, 30 patients received ESD treatment first and were pathologically assessed as noncurative ESD, followed by surgical treatment. The ESD indication was in line with the 5th edition of Japanese gastric cancer treatment guidelines [3]. The ESD noncurative resection was determined as long as the pathology meets one of the following criteria: positive horizontal or vertical margin, vascular infiltration, submucosal infiltration depth ≥500 *μ*m (SM2), differentiated tumor with ulceration (cT1a stage) diameter >3 cm, and the depth of submucosal invasion was <500 *μ*m (SM1), but the diameter was >3 cm; the invasion of the undifferentiated tumor was deep submucosal or larger than 2 cm in diameter or accompanied by ulceration [3]. According to the time of additional surgery, the patients were divided into the early surgery group (≤30 days after ESD, 19 cases) and the delayed surgery group (>30 days after ESD, 11 cases). The other 59 patients underwent upfront surgical treatment.

General data, including age, gender, and body mass index (BMI), were collected. Pathological information consisting of lesion diameter, ulcer, Lauren classification, depth of invasion, vascular invasion, differentiation type, ESD margin, HER-2 expression, residual cancer, and LN metastasis were obtained. For the risk factors of cancer residue and LN metastasis after ESD surgery, the possible influencing factors were analyzed by univariable analysis, and then, the risk factors were obtained by multivariable logistic regressions. Furthermore, the receiver operating characteristic (ROC) curves of the independent factors and the multifactor model were used to judge their predictive capability. To investigate the best timing for additional surgery, intraoperative data including surgery approach, operation time, intraoperative blood loss, number of dissected LN, and postoperative data, containing flatus and defecation time, oral feeding time, postoperative complications, and postoperative hospital stay were compared among three groups (early surgery after ESD, delayed surgery after ESD, and direct surgery).

In this study, SPSS 22.0 (IBM Corp., Armonk, NY, USA) was used for data statistical analysis. Values with a normal distribution were reported as mean ± standard deviation (SD), and skewed data were expressed as median and 25–75% interquartile range (IQR). The difference between groups was compared using the chi-square test or Fisher's exact probability method. *P* was considered statistically significant.

## 3. Results

A total of 89 patients with early stage gastric cancer were included in this study, including 30 patients who underwent additional surgery after noncurative ESD and 59 patients who underwent upfront surgery. The effects of varied surgical time were compared in the overall patients after the noncurative ESD patients were examined.

### 3.1. Basic Characteristics of Patients with Noncurative ESD

Postoperative pathological analysis of 30 patients undergoing additional surgery showed residual cancer in 16 cases and LN metastasis in 5 cases. Patients were divided into two groups according to the presence of cancer residue and LN metastasis, and the differences in basic information and pathological features after ESD were compared between the two groups (Table 1). Between the two groups with and without cancer residue, significant difference in age, Lauren categorization, depth of cancer invasion, horizontal resection margin, vertical resection margin, and human epidermal growth factor receptor-2 (HER-2) expression were found. There were significant differences in tumor differentiation type, vascular invasion, vertical resection margin, and HER-2 expression between patients with and without LN metastasis. There were no significant differences in gender, BMI, ESD indication (absolute or enlarged), ulcer, and diameter between groups with or without cancer residue and with or without LN metastasis.

### 3.2. Risk Factor Analysis of Noncurative ESD Patients with Residual Cancer and LN Metastasis

First, univariable analysis was conducted for the risk factor of residual cancer and LN metastasis. Further multivariable regression analysis showed that diffuse type of Lauren classification (OR = 2.28, 95% CI: 1.81–2.45, *P*=0.014), submucosal invasion (OR = 1.87, 95% CI: 1.32–2.14, *P*=0.023), and positive HER-2 (OR = 2.41, 95% CI: 2.03–2.71, *P*=0.008) were independent risk factors for residual cancer (Table 2). Pathologically undifferentiated (OR = 2.76, 95% CI: 1.87–3.21, *P*=0.021), vascular invasion (OR = 2.53, 95% CI: 2.21–2.98, *P*=0.013), and positive vertical margin (OR = 1.81, 95% CI: 1.65–2.13, *P*=0.027) were independent risk factors for LN metastasis (Table 3). Positive HER-2 was not an independent risk factor for LN metastasis, and age was not an independent risk factor for residual cancer.

### 3.3. The Single Independent Factor and Multifactor Model Predicting Residual Cancer and LN Metastasis

**Figure 1:**
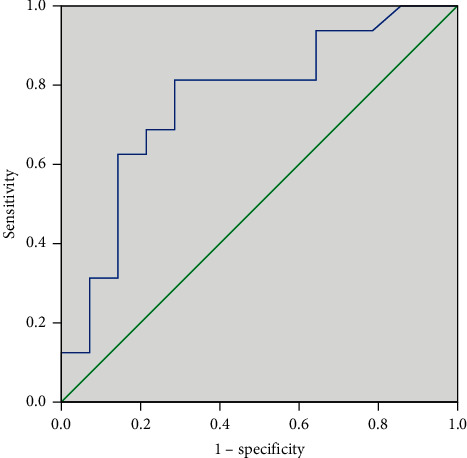
ROC curve for the selected logistic regression model in the diagnosis of residual cancer. Area under the curve = 0.761; sensitivity = 81.3%; specificity = 71.4%.

**Figure 2:**
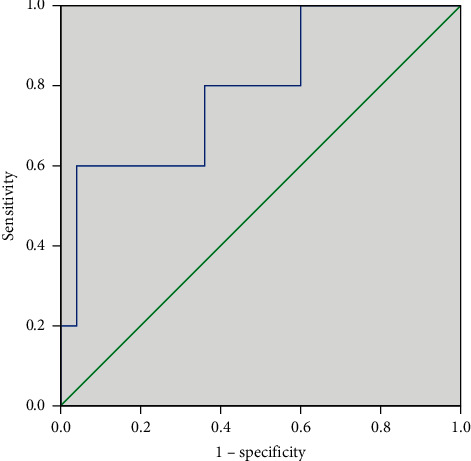
ROC curve for the selected logistic regression model in the diagnosis of LN metastasis. Area under the curve = 0.792; sensitivity = 80.0%; specificity = 64.0%.

The above independent risk factor and multivariable models created ROC curves to assess the accuracy of residual cancer and LN metastatic prediction (Table 4). The results showed that the area under the curve (AUC) predicting cancer residue by the multifactor model was 0.761, the specificity was 0.714, and the sensitivity was 0.813 (Figure 1). The AUC, specificity, and sensitivity of the multifactor model for predicting LN metastasis were 0.792, 0.800, and 0.640 (Figure 2).

### 3.4. Comparison of Additional Surgery with Different Timing after ESD and Direct Surgery in Early Gastric Cancer Patients

In order to determine the optimal timing for additional surgery after noncurative ESD, patients undergoing additional surgery were divided into the early surgery group (≤30 days) and delayed surgery group (>30 days). Patients with early gastric cancer who underwent direct surgery were set as the control group. The safety and radical resection of the three groups were compared. The delayed surgery group had less intraoperative blood loss and a shorter operation time than the early surgery group, according to the findings. There were no significant differences in the number of LN dissections, LN metastatic rate, or postoperative complications between the two groups. The early surgery group also had more intraoperative blood loss and longer hospital stays than the direct surgery group. However, there was no significant difference in intraoperative and postoperative indicators between the delayed surgery group and the direct surgery group (Table 5).

## 4. Discussion

How to accurately predict residual cancer and LN metastasis after noncurative ESD is of great significance to guide clinical practice. There is no clear consensus on the timing of remedial surgery after ESD. Therefore, this study first analyzed the risk factors of cancer residue and LN metastasis in patients with noncurative ESD and then further compared the influence of early and delayed surgery on perioperative safety and radical resection. Postoperative pathologically confirmed tumor residue was found in 18 cases (accounting for 60.0%) of the 30 patients with early gastric cancer who underwent noncurative ESD and additional surgery, including 16 cases with primary tumor residue (accounting for 53.3%) and 5 cases with LN metastasis (accounting for 16.7%). Three of them (10%) had primary residual cancer with LN metastasis. This group of data shows that innocent surgical patients accounted for 40%.

Multivariable regression analysis showed that diffuse type of Lauren classification, submucosal invasion, and positive HER-2 were risk factors for residual cancer. The diffused invasive growth pattern of tumors may hinder endoscopists from accurately determining tumor tissue boundaries. The increased depth of vertical infiltration has the potential to exceed the excision layer of ESD operation. Sangjeong et al. found that the increase of positive margin length was an important risk factor for residual cancer. The sensitivity of positive margins with a total length of more than 6 mm to residual cancer diagnosis was 85.7% [10]. Sunagawa et al. found that positive horizontal and vertical margins were risk factors for residual cancer by analyzing 200 cases of noncurative ESD surgery [11]. Nie et al. observed that tumor diameter >3 cm, undifferentiated type, and positive horizontal margin enhanced the probability of residual cancer in a meta-analysis of 4870 cases [12]. A positive edge means that there are tumor cells within 2 mm of the boundary tissue [13], which is related to the burning of the edge and the fixation of the specimen. Proper surgery and specimen processing can help forecast the likelihood of residual cancer with more accuracy. In addition, endoscopic amplification and staining should be performed routinely before ESD to accurately determine the horizontal boundary of lesions. Endoscopic ultrasonography is useful for determining the depth of lesion invasion and identifying instances that are suited for ESD treatment. Numata et al. found that the overall positive rate of horizontal resection margin was 2% (21/1053) in 1053 cases of early gastric cancer undergoing ESD, and the follow-up found that the local recurrence rate was 0.3% (3/1053) in all patients, and the time of local recurrence ranged from 8 to 34 months [13]. Sekiguchi et al. analyzed 77 patients with positive horizontal resection margin after ESD and selected follow-up. They found that the local tumor recurrence rate within 5 years was 11.9%, and more than 6 mm was an effective indicator to predict recurrence [8]. Surgical operation is recommended for patients with positive vertical margin, but there is no unified opinion on whether to perform ESD again or additional surgery for follow-up treatment with positive horizontal margin (eCura-C1) [3], which needs to be determined by patients' specific conditions and hospital operation routine and needs to be confirmed by clinical studies with larger samples.

In this study, the proportion of LN metastasis in patients with noncurative ESD resection was 16.7% (5/30). This is slightly higher than the reported 9.8% incidence of LN metastasis in patients with additional surgery after endoscopic treatment [14]. This may be related to the fact that most of the patients with noncurative ESD in this study were with the extended ESD indications. Multivariable regression analysis showed that undifferentiated tumor, vascular invasion, and positive vertical margin were risk factors for LN metastasis. Undifferentiated gastric cancer includes poorly differentiated adenocarcinoma, signet-ring cell carcinoma, and mucinous adenocarcinoma. Studies have shown that the LN metastasis rate of these three types of early gastric cancer can reach 6.0%–44.4% [15]. Undifferentiated intramucosal carcinoma above 2 cm is not an absolute indication for ESD because of the relatively high probability of LN metastasis [3]. LN metastasis was 6.7 times higher among patients with lymphovascular invasion than in those without, and LN positivity increased significantly with increasing depth of lesion invasion, according to a postoperative histopathological analysis of 3131 patients with early gastric cancer. Meta studies have found that the LN metastasis rate can reach 2.5% when the tumor infiltrates to the 300 *μ*m submucosal layer, which is close to 2.8% when the tumor infiltrates to the 500 *μ*m submucosal layer [16]. The submucosa contains a large number of lymphatic vessels, and tumor cells that infiltrate the submucosa are more likely to spread to LN via these vessels. Japanese guidelines also clearly indicate that additional surgery is imperative when submucosal invasion exceeds 500 *μ*m, or there is undifferentiated carcinoma or vascular invasion (eCura-C2) [3]. This is consistent with our findings.

However, there is no consensus on when to perform surgery after noncurative ESD. Our study found that delayed surgery (>30 days) was associated with less intraoperative bleeding, operative time, and hospital stay than early surgery (≤30 days), and there were no significant differences in complications or radical outcomes. This is consistent with the results of other studies. By analyzing 154 patients undergoing additional surgery after ESD, Kim et al. found that compared with the delayed surgery group (>29 days), the early surgery group (<29 days) had longer operation time and more intraoperative blood loss [17]. There was no significant difference in the tumor recurrence rate between the two groups after additional follow-up [18]. By analyzing 107 cases of additional surgery after ESD, Lee et al. found that patients with an interval of fewer than 24 days and ESD-related ulcers over 4.6 cm had more intraoperative bleeding and longer operation time [19]. Within 4–8 weeks after ESD surgery, local edema, inflammation, and scar formation of gastric wall tissue exist [20]. This could be one of the reasons for the early surgical group's longer operative time and higher intraoperative blood loss. Tissue edema and inflammation may subside after more than a month, reducing the complexity of the surgery. Some studies have found that convergence of gastric mucosa due to scarring caused by ESD operation in the middle and upper stomach can affect the selection of additional surgical methods, and the proportion of distal gastrectomy is significantly reduced [21]. This is also a significant influence in increasing the duration of surgery and the amount of blood loss. But larger, higher-quality studies are needed to determine the best timing of additional surgery. The gender showed some difference between the direct surgery group and additional surgery group. We considered this was due to the small size of the ESD group which has a relatively high male proportion. Since the gastric surgery was performed in the upper abdomen and the BMI was comparable among the three groups, the gender difference theoretically should have little effect on the surgery process and recovery.

This study has some limitations. First, this was a single-center retrospective study, and some ESD cases were with expanded indications, which may have selection bias. Second, due to the small sample size, the power of test of the factor undifferentiated carcinoma was slightly weak. Thus, when we consider the effect of pathological type on LN metastasis in practice, we should take it with caution based on the present data. In addition, although the prediction ability of risk factors on cancer residue and LN metastasis was analyzed by the ROC curve in this study, it has not been verified in a large number of cases. Third, this study mainly observed the perioperative safety of patients, and the long-term prognosis has not been recorded, which needs to be further studied.

## 5. Conclusions

In conclusion, diffuse type of Lauren classification, submucosal invasion, and positive HER-2 were risk factors for residual cancer, while undifferentiated tumor, vascular invasion, and positive vertical margin were risk factors for LN metastasis. In early gastric cancer patients, delaying surgery after ESD (>30 days) improves intraoperative safety without compromising radical resection.

## Figures and Tables

**Table 1:** Characteristics of patients with noncurative ESD.

	Residual cancer		*P*	LN metastasis		*P*
	Yes (*n* = 16)	No (*n* = 14)		Yes (*n* = 5)	No (*n* = 25)	
Gender						
Male	13	12	0.432	4	21	0.124
Female	3	2		1	4	

Age (years)						
≥65	2	7	0.025	1	8	0.592
<65	14	7		4	17	

BMI (kg/m^2^)	23.1 ± 4.2	22.7 ± 3.6	0.032	24.3 ± 4.1	22.3 ± 3.9	0.104

ESD indication						
Absolute	6	4	0.521	2	8	0.221
Enlarged	10	10		3	17	

Lauren type						
Intestinal	2	11	0.001	1	12	0.191
Diffuse	14	3		4	13	

Ulcer						
Yes	5	4	0.523	2	7	0.592
No	9	12		3	18	

Lesion diameter						
≤3 cm	12	10	0.825	4	18	0.712
>3 cm	4	4		1	7	

Pathological type						
Differentiated	7	6	0.964	1	12	0.024
Undifferentiated	9	8		4	13	

Depth of invasion						
Mucosa	2	9	0.003	0	11	0.062
Submucosa (or deeper)	14	5		5	14	

Vascular invasion						
Positive	4	3	0.818	4	3	0.003
Negative	12	11		1	22	

Vertical margin						
Positive	12	0	0.001	4	6	0.015
Negative	4	14		1	19	

Horizontal margin						
Positive	9	0	0.001	2	9	0.864
Negative	7	14		3	16	

HER-2 expression						
Positive	11	1	0.001	4	8	0.045
Negative	5	13		1	17	

**Table 2:** Univariable analysis and multivariable logistic regression analysis for residual cancer.

	Univariable			Multivariable		
	OR	95% CI	*P*	OR	95% CI	*P*
Age < 65	1.21	0.78–1.67	0.109			
Diffuse type of Lauren classification	3.67	2.53–3.78	0.007	2.28	1.81–2.45	0.014
Submucosal invasion	2.12	1.56–2.81	0.019	1.87	1.32–2.14	0.023
HER-2 positive	3.32	2.13–4.02	0.001	2.41	2.03–2.71	0.008

**Table 3:** Univariable analysis and multivariable logistic regression analysis for LN metastasis.

	Univariable			Multivariable		
	OR	95% CI	*P*	OR	95% CI	*P*
Undifferentiated carcinoma	2.82	2.10–3.43	0.015	2.76	1.87–3.21	0.021
Vascular invasion	2.79	1.91–3.27	0.011	2.53	2.11–2.98	0.013
Vertical margin positive	1.97	1.72–2.34	0.021	1.81	1.65–2.13	0.027
HER-2 positive	1.19	0.91–1.44	0.213			

**Table 4:** The single independent factor and multifactor model predicting residual cancer and LN metastasis.

Factors	AUC	Specificity	Sensitivity
Predicting residual cancer			
Multifactor model	0.761	0.714	0.813
Diffuse type of Lauren classification	0.536	0.645	0.595
Submucosal invasion	0.673	0.589	0.829
HER-2 positive	0.553	0.512	0.614

Predicting LN metastasis			
Multifactor model	0.792	0.640	0.800
Undifferentiated carcinoma	0.581	0.504	0.631
Vascular invasion	0.629	0.577	0.812
Vertical margin positive	0.521	0.492	0.587

**Table 5:** Comparison of safety and radical resection between different surgery timing for early gastric cancer.

	Early surgery after ESD (*n* = 19)	Delayed surgery after ESD (*n* = 11)	Direct surgery (*n* = 59)	P1 (early vs. delayed)	P2 (early vs. direct)	P3 (delayed vs. direct)
Age (year)	61.4 ± 10.3	63.5 ± 8.9	62.7 ± 11.4	0.532	0.312	0.571
Gender (male)	17	8	33	0.093	0.012	0.037
BMI (kg/m^2^)	22.1 ± 4.2	24.5 ± 3.9	23.6 ± 4.7	0.211	0.421	0.542
Extent of gastric resection						
Distal gastrectomy	13	8	42	0.533	0.242	0.471
Proximal gastrectomy	2	1	6			
Total gastrectomy	4	2	11			
Surgery approach						
Laparoscopic	12	8	38	0.231	0.123	0.701
Open	7	3	21			
Operation time (min)	289 ± 74	230 ± 66	245 ± 102	0.046	0.072	0.144
Intraoperative blood loss (ml)	421 ± 218	252 ± 102	321 ± 138	0.012	0.025	0.059
No. of LN dissection	22 ± 7	19 ± 8	23 ± 7	0.634	0.453	0.323
LN metastasis rate	15.8% (3/19)	18.2% (2/11)	13.6% (8/59)	0.324	0.279	0.145
Postoperative flatus and defecation time (d)	6.1 ± 2.8	4.8 ± 2.2	4.9 ± 3.1	0.139	0.051	0.231
Postoperative oral feeding time (d)	5.3 ± 3.8	4.7 ± 3.5	4.7 ± 2.9	0.711	0.213	0.572
Postoperative hospital stay (d)	12.3 ± 5.8	10.5 ± 4.1	9.8 ± 2.9	0.062	0.031	0.342
Postoperative complications (*n*)	4	3	12	0.312	0.192	0.211

## Data Availability

The datasets collected and analyzed in this study are available from the corresponding author upon request.
